# Non-alcoholic fatty liver disease (NAFLD), metabolic syndrome and cardiovascular events in atrial fibrillation. A prospective multicenter cohort study

**DOI:** 10.1007/s11739-021-02682-3

**Published:** 2021-03-13

**Authors:** Daniele Pastori, Angela Sciacqua, Rossella Marcucci, Maria Del Ben, Francesco Baratta, Francesco Violi, Pasquale Pignatelli, the ATHERO-AF study group, Mirella Saliola, Danilo Menichelli, Marco Antonio Casciaro, Francesco Angelico, Vittoria Cammisotto, Cristina Nocella, Simona Bartimoccia, Roberto Carnevale, Laura Novelli

**Affiliations:** 1 I Clinica Medica, Atherothrombosis Centre, Department of Clinical Internal, Anaesthesiological and Cardiovascular Sciences, Sapienza University of Rome, Viale del Policlinico 155, 00161 Rome, Italy; 2 Department of Medical and Surgical Sciences, University Magna Græcia of Catanzaro, Catanzaro, Italy; 3 Department of Experimental and Clinical Medicine, University of Florence, Florence, Italy; 4 Mediterranea Cardiocentro, Naples, Italy

**Keywords:** Atrial fibrillation, NAFLD, Metabolic syndrome, Cardiovascular events

## Abstract

Whether non-alcoholic fatty liver disease (NAFLD) is associated with an increased risk of cardiovascular events (CVEs) independently from metabolic syndrome (MetS) is still matter of debate. Aim of the study was to investigate the risk of CVEs in a high-risk population of patients with non-valvular atrial fibrillation (AF) according to the presence of MetS and NAFLD. Prospective observational multicenter study including 1,735 patients with non-valvular AF treated with vitamin K antagonists (VKAs) or direct oral anticoagulants (DOACs). NAFLD was defined by a fatty liver index ≥ 60. We categorized patients in 4 groups: 0 = neither MetS or NAFLD (38.6%), 1 = NAFLD alone (12.4%), 2 = MetS alone (19.3%), 3 = both MetS and NAFLD (29.7%). Primary endpoint was a composite of CVEs. Mean age was 75.4 ± 9.4 years, and 41.4% of patients were women. During a mean follow-up of 34.1 ± 22.8 months (4,926.8 patient-years), 155 CVEs were recorded (incidence rate of 3.1%/year): 55 occurred in Group 0 (2.92%/year), 12 in Group 1 (2.17%/year), 45 in Group 2 (4.58%/year) and 43 in Group 3 (2.85%/year). Multivariable Cox regression analysis showed that use of DOACs, and female sex were inversely associated with CVEs, whilst age, heart failure, previous cardiac and cerebrovascular events, and group 2 (Group 2, Hazard Ratio 1.517, 95% Confidence Interval, 1.010–2.280) were directly associated with CVEs. In patients with AF, MetS increases the risk of CVEs. Patients with NAFLD alone have lower cardiovascular risk but may experience higher liver-related complications.

## Introduction

Patients with atrial fibrillation (AF) are characterized by a high atherosclerotic burden, as demonstrated by the high prevalence of multiple atherosclerotic risk factors, such as arterial hypertension, diabetes, obesity and dyslipidemia, accounting for an increased risk of myocardial infarction (MI) in this population [1, 2]. All these factors are usually clustered to define the metabolic syndrome (MetS) [3, 4], a condition characterized by a high risk of cardiovascular events (CVEs) [5]. Previous studies showed that MetS is highly prevalent in patients with AF, with almost 40% of patients being affected by this condition [6]. Noteworthy, patients with AF and MetS disclose an increased risk of CVEs as compared to those without [7].

Non-alcoholic fatty liver disease (NAFLD) is the most common liver disease worldwide and has been long regarded as the hepatic manifestation of MetS [8]. NAFLD may range from simple steatosis to steatohepatitis carrying an increased risk of liver cirrhosis and hepatocellular carcinoma and representing a common indication to liver transplantation [9]. Some reports showed that patients with NAFLD have an high rate of cardiovascular comorbidities, resulting in an increased cardiovascular risk [10, 11]. Thus, in patients with NAFLD, an increased coronary artery calcification as well as abnormalities in cardiac function have been described, leading to an increased risk of cardiac arrhythmias including AF [12]. However, not all NAFLD are of metabolic origin, and some NAFLD patients do not present with the features of MetS. The cardiovascular risk of these patients is unclear, as well as if NAFLD should be considered per se a cardiovascular risk factor (independently from MetS) is still a matter of debate.

In a recent study, we found that the prevalence of NAFLD, as assessed by the fatty liver index (FLI) was as high as 42% [13]. Despite this high prevalence, the presence of NAFLD was not associated with ischemic or bleeding risk [13].

However, the relationship of NAFLD with the presence of MetS in AF has not been investigated. In this analysis, we describe the risk of CVEs in AF patients according to the presence of NAFLD and MetS, alone or in combination.

## Methods

Details of this cohort have been previously reported [13]. Briefly, 1,735 outpatients with non-valvular AF (defined by absence of mechanical prosthetic heart valve or moderate–severe mitral stenosis) on treatment with oral anticoagulants, both vitamin K antagonists (VKAs) and direct oral anticoagulants (DOACs) have been included in this study. Patients were recruited from three centers of internal medicine and cardiology in Rome, Catanzaro and Florence, Italy. NAFLD was diagnosed by the validated fatty liver index (FLI) with a cut-off > 60; MetS was defined by the modified ATP-III criteria, as previously described [13].

Principal endpoint was the occurrence of CVEs, defined by a composite of fatal/non-fatal myocardial infarction or ischemic stroke, transient ischemic attack (TIA) and cardiovascular death.

### Statistical analysis

Categorical variables were reported as counts (percentages). The normal distribution of parameters was assessed by Kolmogorov–Smirnov test. Continuous variables were expressed as mean ± standard deviation. Student *t* test for unpaired samples was used to compare means. Independence of categorical variables was tested with the *χ*^2^ test.

Patients were divided into four groups as follows: 0 = neither MetS nor NAFLD, 1 = NAFLD alone, 2 = MetS alone, 3 = both MetS and NAFLD. Characteristics of patients according to each study group have been described. Univariate and multivariate Cox proportional hazards regression analysis was used to calculate the relative adjusted hazard ratio (HR) and 95% confidence interval (CI) for CVEs by each clinical variable. In addition to the four study groups, the following covariates were entered in the multivariable model: type of oral anticoagulant (DOAC vs VKAs), persistent/permanent AF (vs paroxysmal), sex, age (continuous), current cigarette smoking, heart failure (HF), previous cardiac and cerebrovascular events, eGFR, antiplatelet drug, statin, digoxin. Arterial hypertension, diabetes and obesity indices were not included as already present in the MetS and FLI variables.

Statistical significance was set at a *p* value < 0.05. All tests were two-tailed and analyses were performed using computer software packages (SPSS-25.0, SPSS Inc.).

The study protocol was approved by the local ethical board of Sapienza-University of Rome and was conducted according to the principles of the Declaration of Helsinki.

## Results

Overall, 669 (38.6%) AF patients were not diagnosed with MetS or NAFLD (Group 0), 216 (12.4%) had NAFLD alone (without MetS, Group 1) and 334 (19.3%) had MetS alone (without NAFLD, Group 2). MetS and NAFLD coexisted in 516 (29.7%) of patients (Group 3). Table 1 reports clinical and biochemical characteristics of each group. The group of patients with NAFLD alone disclosed the lowest proportion of women, and a low prevalence of cardiovascular risk factors, such as hypertension, diabetes and heart failure; conversely, these patients presented with elevated values of GGT (Table 1). Patients with MetS showed the highest mean age and proportion of women, while they were less likely to be treated with DOACs (Table 1).

**Table 1:** Clinical and biochemical characteristics of atrial fibrillation patients according to the presence of metabolic syndrome and NAFLD

	Group 0 (No MetS, No NAFLD) n = 669	Group 1 (NAFLD alone) n = 216	*p* value (1 vs group 0)	Group 2 (MetS alone) n = 334	*p* value (2 vs group 0)	*p* value (2 vs group 1)	Group 3 (MetS + NAFLD) n = 516	*p* value (3 vs group 0)	*p* value (among groups)
Age (years)	75.4 ± 9.4	73.5 ± 9.9	0.010	76.2 ± 8.1	0.186	0.001	73.8 ± 8.7	0.004	< 0.001
Women (%)	41.4	23.1	< 0.001	63.8	< 0.001	< 0.001	42.8	0.635	< 0.001
DOAC (vs. VKA) (%)	57.2	64.8	0.056	54.5	0.418	0.017	62.6	0.065	0.024
Persistent/permanent AF (%)	53.4	56.9	0.388	62.0	0.010	0.248	65.7	< 0.001	< 0.001
Body Mass Index *(Kg/m*^*2*^*)*	24.2 ± 2.9	29.7 ± 4.1	< 0.001	25.6 ± 2.4	< 0.001	< 0.001	31.3 ± 4.2	< 0.001	< 0.001
Waist circumference (cm)	91.8 ± 8.4	107.3 ± 9.9	< 0.001	95.2 ± 7.4	< 0.001	< 0.001	110.1 ± 9.8	< 0.001	< 0.001
GGT (U/l)	29.5 ± 19.4	51.9 ± 39.5	< 0.001	25.9 ± 15.2	0.040	< 0.001	43.9 ± 33.3	< 0.001	< 0.001
Triglycerides (mg/dl)	98.3 ± 36.7	119.4 ± 50.2	< 0.001	116.1 ± 49.4	< 0.001	0.504	161.5 ± 80.6	< 0.001	< 0.001
Total cholesterol (mg/dl)	182.4 ± 43.0	193.4 ± 44.5	0.003	173.4 ± 47.4	0.004	< 0,001	186.1 ± 52.4	0.175	< 0.001
HDL (mg/dl)	53.8 ± 15.8	54.4 ± 16.4	0.629	44.0 ± 15.1	< 0.001	< 0.001	45.0 ± 16.4	< 0.001	< 0.001
LDL (mg/dl)	109.4 ± 37.3	116.4 ± 42.6	0.030	106.9 ± 41.2	0.379	0.009	111.4 ± 45.2	0.398	0.054
Current smoking (%)	7.9	7.9	0.980	9.9	0.338	0.452	10.5	0.152	0.402
GFR (ml/min/1.73 m^2^)	75.9 ± 22.5	75.2 ± 20.8	0.689	71.4 ± 22.6	0.003	0.053	72.1 ± 22.4	0.003	0.003
HAS-BLED score	1.6 ± 0.9	1.6 ± 0.8	0.556	1.6 ± 0.9	0.791	0.746	1.5 ± 0.9	0.057	0.277
CHA_2_DS_2_ VASc score	3.4 ± 1.5	3.1 ± 1.4	0.114	4.3 ± 1.4	0.098	0.127	3.8 ± 1.5	0.085	< 0.001
Arterial Hypertension (%)	78.6	80.6	0.565	98.2	< 0.001	< 0.001	95.9	< 0.001	< 0.001
Diabetes (%)	9.3	7.4	0.490	35.6	< 0.001	< 0.001	41.1	< 0.001	< 0.001
Heart failure (%)	14.8	12.0	0.369	20.4	0.031	0.015	17.8	0.176	0.032
Previous cerebrovascular events (%)	14.9	15.7	0.827	19.2	0.103	0.361	17.8	0.203	0.315
Previous cardiac events (%)	16.0	20.8	0.119	24.3	0.002	0.406	21.5	0.016	0.010
Antiplatelet drugs (%)	12.0	16.2	0.130	11.4	0.836	0.122	12.4	0.858	0.351
Insulin (%)	1.2	1.9	0.469	6.3	< 0.001	0.019	9.1	< 0.001	< 0.001
Statins (%)	38.9	39.8	0.810	49.4	0.002	0.029	51.6	< 0.001	< 0.001
Digoxin (%)	16.7	14.4	0.457	17.7	0.722	0.346	15.3	0.525	0.678

*AF* atrial fibrillation, *DOAC* direct oral anticoagulants, *GFR* glomerular filtration rate, *GGT* gamma-glutamyl transpeptidase, *HDL* high-density lipoprotein, *LDL* low-density lipoprotein, *MetS* metabolic syndrome, *NAFLD* non-alcoholic fatty liver disease, *VKA* vitamin K antagonist

Patients with MetS and NAFLD were more frequently affected by persistent/permanent AF, with the highest degree of obesity and prevalence of diabetes compared to the other groups (Table 1). Nearly half of the patients in groups 2 and 3 was on treatment with statins.

### Cardiovascular events

**Fig. 1:**
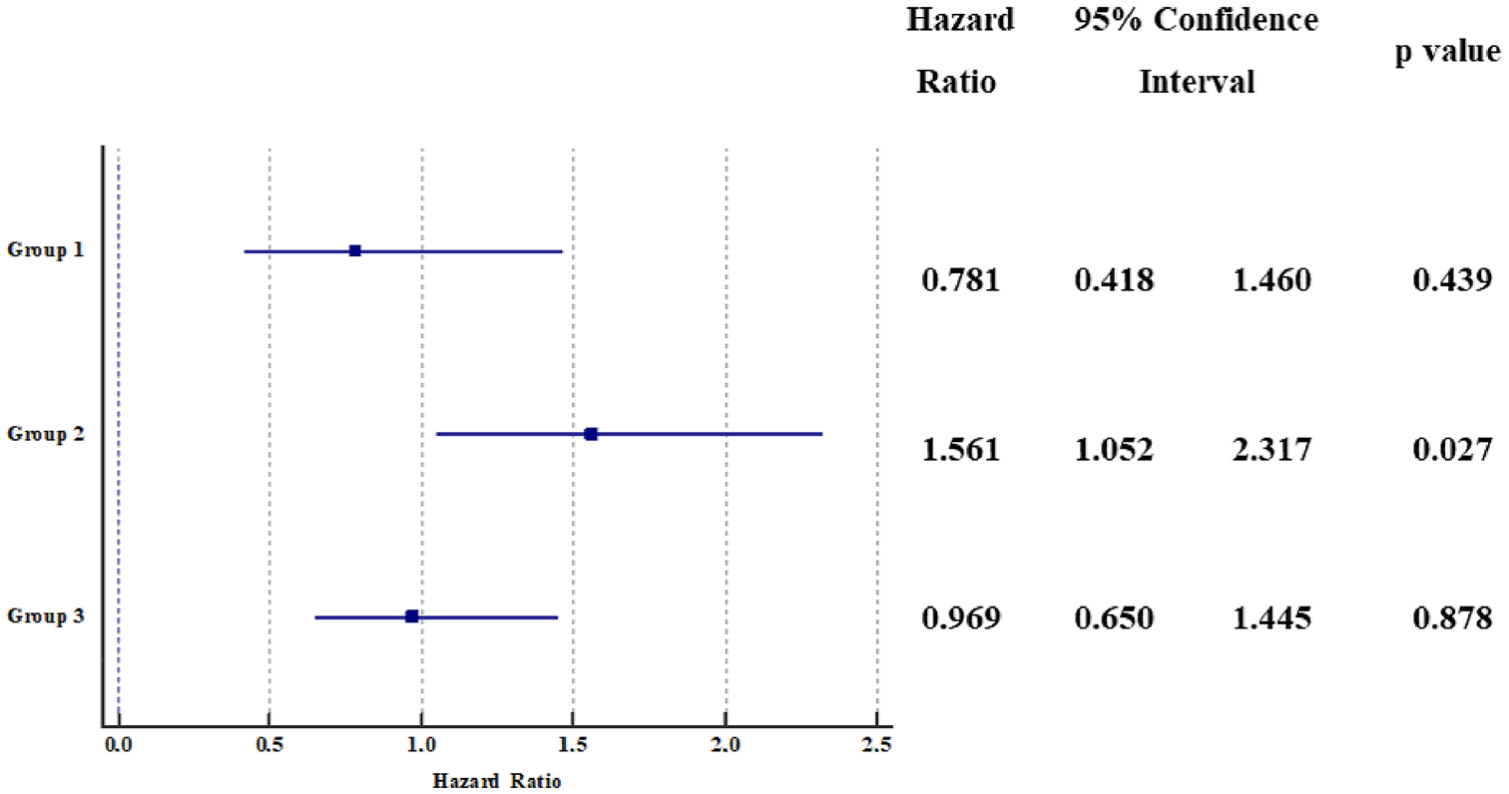
Univariate hazard ratio by each group for cardiovascular events

**Fig. 2:**
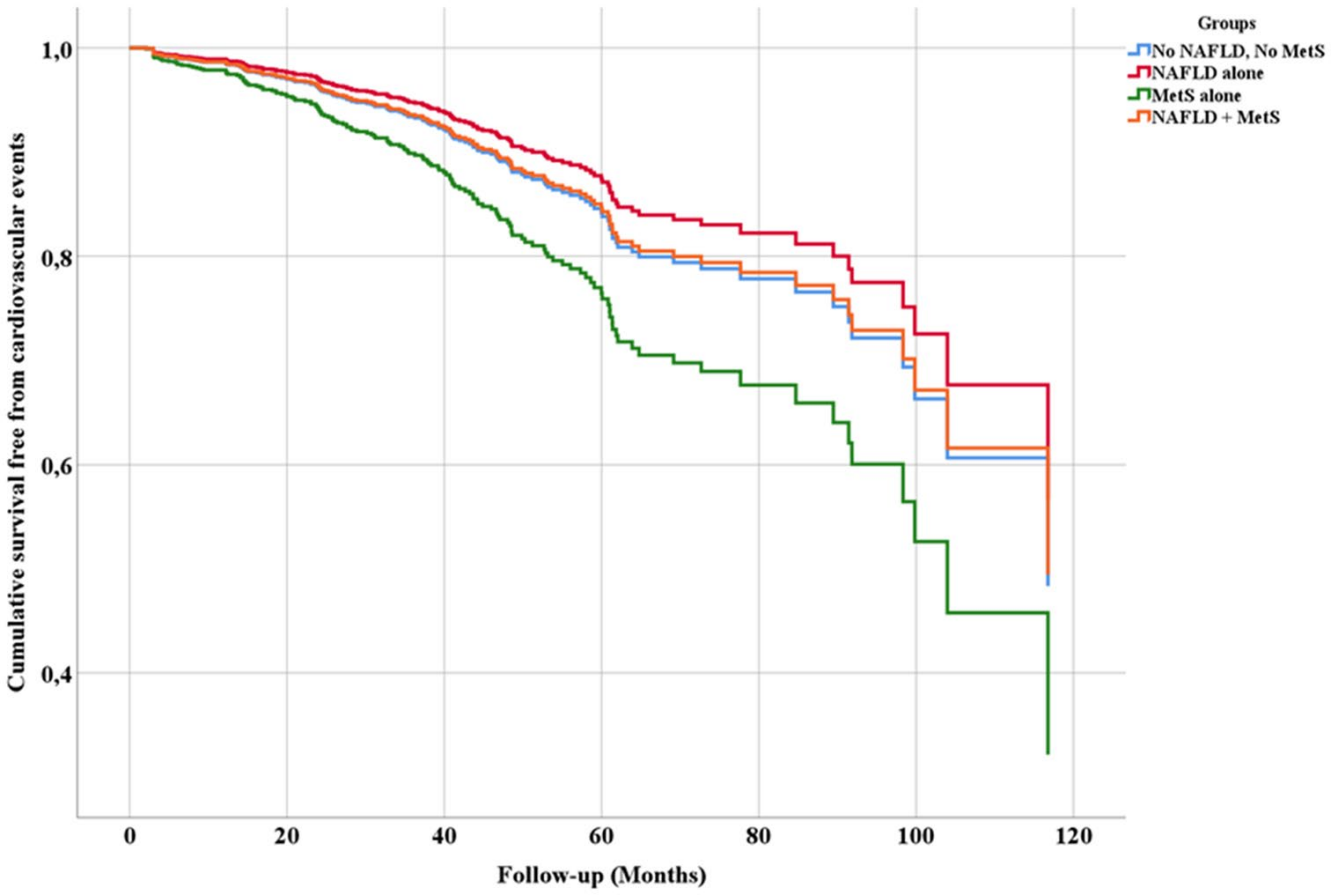
Adjusted cumulative survival curves according to each study group

During a mean follow-up of 34.1 ± 22.8 months (4,926.8 patient-years), 155 CVEs were recorded with an incidence rate (IR) of 3.15%/year (95% CI 2.67–3.68): 55 occurred in Group 0 (2.92%/year, 95% CI 2.20–3.80), 12 in Group 1 (2.17%/year, 95% CI 1.12–3.79, p = 0.352 vs. group 0), 45 in Group 2 (4.58%/year, 95% CI 3.34–6.13, *p* = 0.024 vs. group 0) and 43 in Group 3 (2.85%/year, 95% CI 2.06–3.84, *p* = 0.909 vs. group 0). Figure 1 shows univariate HR by each study group. At multivariable Cox proportional hazard regression analysis (Table 2), we found that use of DOACs (vs VKAs), and female sex were inversely associated with the risk of CVEs, whilst age, heart failure, previous cardiac and cerebrovascular events, and group 2 (MetS alone) were directly associated with CVEs. This was also displayed by adjusted survival curves (Fig. 2), showing a decreased survival for group 2 compared to other groups.

**Table 2:** Cox proportional hazard regression analysis of factors associated with cardiovascular events

	*p* value	Hazard ratio	95% confidence interval	
DOACs (vs VKAs)	0.012	0.621	0.428	0.901
Persistent/permanent AF	0.410	0.868	0.620	1.215
Female sex	0.009	0.623	0.437	0.889
Age (continuous)	0.000	1.064	1.039	1.090
Current cigarette smoking	0.063	1.606	0.975	2.644
Heart Failure	0.004	1.727	1.189	2.507
Previous cardiac events	0.008	1.658	1.143	2.406
Previous cerebrovascular events	0.000	2.224	1.546	3.200
GFR (continuous)	0.145	1.005	0.998	1.012
Antiplatelet drugs	0.500	1.168	0.745	1.831
Group 1 (NAFLD alone)*	0.890	0.956	0.504	1.811
Group 2 (MetS alone)*	0.045	1.517	1.010	2.280
Group 3 (NAFLD + MetS)*	0.551	1.133	0.751	1.709
Statins	0.388	0.862	0.616	1.207
Digoxin	0.193	1.298	0.876	1.922

*AF* atrial fibrillation, *DOACs* direct oral anticoagulants, *GFR* glomerular filtration rate, *MetS* metabolic syndrome, *NAFLD* non-alcoholic fatty liver disease, *VKA* vitamin K antagonist

^*^Group 0 (No MetS, no NAFLD) as reference group. Global *p* value *p* = 0.209

## Discussion

Our study shows that the cardiovascular risk in patients with AF seems to be driven by the presence of MetS, while the coexistence of NAFLD does not seem to modify the association with CVEs.

The relationship between NAFLD, with or without MetS, and cardiovascular risk has been studied in few previous works in non-AF patients. Younossi et al. in a cohort of 6,709 patients from the National Health and Nutrition Examination Survey III (NHANES III) database, of whom 1,448 with hepatic steatosis at ultrasound, analyzed the relationship between NAFLD and CVEs. The age of patients was relatively young with a mean age of 50 years; the majority of NAFLD patients (78.9%) had also MetS. In patients who presented with association of NAFLD and MetS, the risk of total (*p* < 0.001), and cardiovascular mortality (p < 0.001), was increased [14]. This result was not evident in patients with NAFLD alone [14].

A study by Karajamaki et al. that included 958 middle-aged people, showed that the risk of CVEs was higher in patients with MetS, with or without NAFLD [15].

However, all these studies were performed in a middle-aged population, at low cardiovascular risk and not affected by AF. Our study included a population of elderly patients with a mean age of 75 years, characterized by a high prevalence of cardio-metabolic diseases, such hypertension, diabetes and obesity.

The clinical phenotype of patients with NAFLD alone in our study, characterized by a lower prevalence of cardiovascular comorbidities, lower use of insulin, and high GGT values, suggest that these patients may have a liver steatosis not strictly correlated to metabolic abnormalities, but rather to a genetic predisposition. These patients may benefit from a genetic screening for PNPLA3 mutation, to identify those with a more aggressive disease and at higher risk of progression to liver cirrhosis [16]. Furthermore, patients with NAFLD alone were less likely to be women, confirming a high prevalence of NAFLD in males than females also in AF patients [4]. A longer follow-up will clarify if this group of patients will experience more liver-related complications than CVEs compared to those affected by MetS.

The data of our work also demonstrates in a population affected by AF that the relationship between metabolic diseases and cardiovascular risk is strongly linked to the presence of MetS, rather than to NAFLD itself. This result highlights the need for a comprehensive management of patients with AF, which should consist in addressing potential modifiable risk factors, such as obesity, and in reaching optimal guideline-recommended targets for blood pressure and glucose control. Thus, in patients with AF, an optimal management of comorbidities according to the Atrial fibrillation Better Care (ABC) pathway (A, Avoid stroke with anticoagulation; B, better symptom management; C, Cardiovascular and comorbidity risk management) resulted in a lower CVEs incidence [17, 18].

Another interesting result of our study is that the treatment with DOAC, compared to VKA, is associated with a reduction of the risk of cardiovascular events. Of note, the use of DOACs was lower in patients with MetS, which may depend on the fact that the use of DOAC in morbid obese patients is still uncertain [19].

Limitations of the study include enrollment of elderly Caucasian patients only, so the application of these data to patients of other ethnicities is uncertain. Furthermore, the observational study design does not allow to establish a cause–effect relationship between the studied variables and the observed endpoints. However, results come from a multicenter prospective study with a structured follow-up. Another limitations is represented by the lack of data on blood glucose control, which may affect cardiovascular risk.

In conclusion, in patients with AF, MetS seems to be the main factor leading to incident CVEs, independently from the presence of NAFLD. Patients with NAFLD alone showed lower cardiovascular risk, but a longer follow-up will clarify if these patients are more likely to experience liver-related complications than CVEs.
